# An outbreak investigation of *Burkholderia cepacia* infections related with contaminated chlorhexidine mouthwash solution in a tertiary care center in Turkey

**DOI:** 10.1186/s13756-021-01004-8

**Published:** 2021-10-10

**Authors:** Hüseyin Bilgin, Gülşen Altınkanat Gelmez, Fatma Bayrakdar, Elvan Sayın, Fethi Gül, Nazlı Pazar, Gülcan Çulha, Serap Süzük Yıldız, Ismail Cinel, Volkan Korten

**Affiliations:** 1 Infectious Diseases, and Clinical Microbiology, Marmara University Hospital, Istanbul, Turkey; 2 Medical Microbiology, Marmara University Hospital, Istanbul, Turkey; 3 National Molecular Microbiology Reference Laboratory, Public Health General Directorate, Ankara, Turkey; 4 Anesthesiology & Reanimation, and Critical Care, Marmara University Hospital, Istanbul, Turkey; 5 Infection Prevention and Control, Marmara University Hospital, Istanbul, Turkey; 6 Marmara University Hospital, Fevzi Cakmak Mah, Muhsinyazicioglu Cad No: 10 Pendik, Istanbul, Turkey

**Keywords:** *Burkholderia cepacia*, Outbreak, Intensive care unit, Chlorhexidine mouthwash, Ventilator-associated-pneumoniae, Healthcare associated infection, Contamination, Outbreak investigation

## Abstract

**Background:**

We report a nosocomial outbreak caused by *Burkholderia cepacia* that occurred among six patients admitted in the medical and surgical intensive care unit between 04 March 2019 and 02 April 2019 in Istanbul, Turkey.

**Methods:**

The outbreak investigation was launched on 11 March 2019 five days after the detection of *B. cepacia* in four different patients. We defined potential reservoirs and started environmental screening. We sampled the liquid solutions used in patient care activities. Pulse-field gel electrophoresis (PFGE) was performed to determine the genetic relatedness of environmental and patient samples.

**Results:**

*Burkholderia cepacia* was isolated in tracheal aspiration cultures of six patients. Three out of six patients developed healthcare-associated pneumoniae due to *B. cepacia*. Environmental cultures in the ICUs revealed *B. cepacia* growth in 2% chlorhexidine-gluconate mouthwash solution that been used in the colonized patients as well as in samples obtained from the unused products. PFGE revealed the patient and a specific batch of chlorhexidine mouthwash solution samples had a 96% similarity.

**Conclusion:**

Contamination of medical solutions used in critical patient care could cause outbreaks and should be detected early by infection control teams.

**Graphic abstract:**

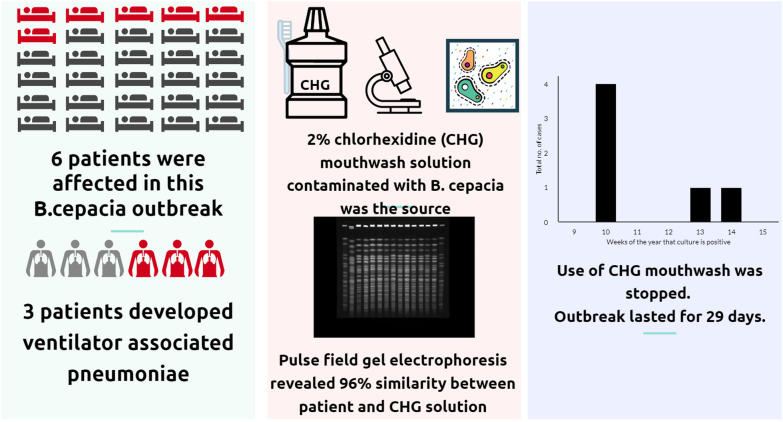

## Introduction

*Burkholderia cepacia* is an aerobic, gram-negative, non-fermentative bacteria frequently isolated from water and solutions [1]. It can survive in fluids, antiseptic solutions, and the healthcare environment for long durations. *Burkholderia cepacia* complex is significant in cystic fibrosis patients, patients with structural lung diseases, and immunocompromised patients [2]. A variety of sources causes *B. cepacia* outbreaks.

Previous reports have demonstrated transmission from contaminated liquids or moist environmental surfaces in hospital settings [3–5]. Here we describe a nosocomial outbreak caused by *Burkholderia cepacia* between 04 March and 02 April 2019 in a tertiary care center in Istanbul, Turkey. The outbreak occurred in patients admitted in intensive care units (ICU) with and without structural lung disease. We report the identification of the source as contaminated 2% chlorhexidine (CHG) mouthwash solution.

## Methods

### Setting

The hospital is a tertiary care center with 659 beds in total, which have 80 ICU beds. The outbreak occurred in the intensive care unit. The ICU is a third level facility, giving care to surgical, trauma, and immunosuppressed patient populations.

The infection prevention and control (IPC) team performs active surveillance of hospital-acquired infections (HAI) in all ICUs in the hospital. IPC nurses and doctors work with the ICU and microbiology team when detecting HAIs. Centers for Disease Control (CDC) definitions were used for HAI diagnosis [6]. Clinical and active surveillance cultures are checked daily, and the IPC team runs daily rounds in the ICU. IPC runs the outbreak investigation in cooperation with the microbiology department.

### Outbreak investigation and environmental sampling

The outbreak investigation was launched on 11 March 2019 five days after the detection of *B. cepacia* in respiratory cultures of four different patients. These patients were discussed in the infection control committee. The IPC team performed a field investigation. We defined potential reservoirs and started environmental screening. We sampled the liquid solutions used in patient care, such as chlorhexidine soap, chlorhexidine mouth wash, and ultrasound gel, intubation, ventilation, and oxygenation equipment. We took samples from sink drains in the patient care rooms. Hands of 6 healthcare workers (HCW) were cultured.

Cultures collected from patients and environment, inoculated on plates and incubated at 36 °C for 18–24 h. Blood, MacConkey and chocolate agar base were used, selective agar plates were not used for *B. cepacia*. Growth of colonies was detected according to the manufacturer’s instructions, and colony species were identified by the VITEK-MS® system (bioMérieux SA, Marcy l’Etoile, France). Antibiotic susceptibility were determined by disk diffusion test and gradient strip test (E-test, bioMérieux).

Pulsed-field gel electrophoresis (PFGE) was done according to the CDC protocol (7). Dendrograms and cluster analysis were generated using the Bionumerics 7.5 (Applied Maths) program with the Unweighted Pair Group Method with the mathematical average (UPGMA) method and the Dice similarity coefficient. *Salmonella braenderup* ATCC BAA664 isolate was used for molecular size indicator [7].

## Results

### Information regarding the patient charactersitcs is presented in Table 1

Patient 1: A male patient who is 75 years-old with chronic renal disease and myasthenia gravis transferred from a long-term care facility with respiratory failure on 21 February 2019. On day eleven of admission, *B. cepacia* was isolated from a tracheal aspiration sample. The patient was diagnosed with ventilator-associated pneumonia (VAP) due to *B. cepacia*, and treatment with piperacillin–tazobactam was started and continued for ten days. The patient improved, but the patient deceased on admission day 55.

**Table 1:** Characteristics of patients with *B. cepacia* colonization/infection

Patient #	Age, year	Gender	Comorbidities	Admission diagnosis	VAP due to *B. cepacia*	Secondary infection	LOS to *B. cepacia* isolation	LOS in ICU	Outcome
1	75	Male	Chronic renal failure, myasthenia gravis	Respiratory failure	Yes on 11^th^ day	MRSA pneumonia	11	55	Deceased
2	85	Male	Alzheimer’s	Community acquired pneumonia	No	Acinetobacter baumannii VAP	22	95	Deceased
3	86	Female	Sigmoid adenocarcinoma	Intraabdominal sepsis	No	No	2	23	Discharged alive
4	69	Male	Diabetes	Myocardial infarction	Yes on 8^th^ day	No	8	45	Deceased
5	73	Male	Chronic renal failure, chronic obstructive pulmonary disease	Community acquired pneumonia	Yes on 60^th^ day	No	60	130	Deceased
6	77	Male	Cervical spinal stenosis	Septic shock	No	Acinetobacter baumannii pneumonia	56	56	Deceased

Patient 2: A 85 years-old male patient with Alzheimer's was admitted with community-acquired pneumonia to another healthcare facility. Later he was transferred to the ICU. Respiratory samples grew *B. cepacia* on day 21 of admission. The patient did not fulfill the VAP criteria. On admission day 80, the patient developed VAP due to *Acinetobacter baumannii* and died on day 95.

Patient 3: A 86 years-old female patient with sigmoid adenocarcinoma underwent re-laparotomy due to an anastomose leak. The patient was diagnosed with intraabdominal sepsis and treated with piperacillin–tazobactam and vancomycin. On admission day two, *B. cepacia* was detected in the tracheal aspirate. Antimicrobial treatment for intraabdominal sepsis was streamlined to piperacillin–tazobactam and levofloxacin. The patient recovered from intraabdominal infection and was discharged to the surgical ward on admission day 23 and eventually discharged from the hospital on day 25.

Patient 4: A male patient who is 69 years-old with diabetes was admitted with subacute anterior myocardial infarction to the intensive care unit. He underwent left anterior descendent artery stent placement. The patient was intubated due to respiratory insufficiency. On admission day eight, *B. cepacia* was isolated from respiratory cultures, and the patient was diagnosed with VAP. Cefepime was started then de-escalated to levofloxacin. He received 14 days of antimicrobial treatment. The patient died on admission day 45.

Patient 5: A male patient who is 73 years-old with chronic obstructive pulmonary disease and chronic renal failure was admitted to the ICU on 24 January 2019. The patient was diagnosed with community-acquired pneumonia and treated with ceftriaxone and clindamycin. *B. cepacia* was isolated from tracheal aspirate on admission day 60. Levofloxacin treatment was started and continued for ten days. The patient was deceased on admission day 130.

Patient 6: A male patient who is 77 years-old, operated on 21 January 2019 for cervical spinal stenosis. The patient was admitted to ICU with septic shock on 05 February 2019. On day 56 of admission to ICU, *B. cepacia* was isolated from deep tracheal aspirate culture. The patient died on the same day. Retrospective examination showed death was not related to a HAI due to *B. cepacia.*

**Fig. 1:**
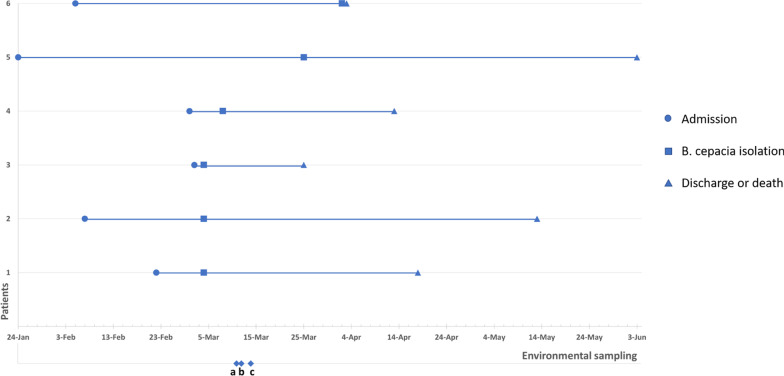
Timeline of six patients who were admitted to the intensive care unit and had *Burkholderia cepacia* detected in respiratory specimens during inpatient treatment. Environmental sampling: **a** mouthwash solution of colonized patients and remaining environmental samples, **b** mouthwash solutions of all patients in the unit and unopened stock solutions in the unit, **c** different batches of unopened mouthwash solutions in the storage area

The six patients' timeline between admission, detection of *B. cepacia*, and discharge/death is displayed in Fig. 1.

In the beginning of the outbreak investigation, we took 34 environmental samples and six hand cultures from HCWs. *B. cepacia* was detected in opened mouthwash products in the unit. Once the growth detected in the mouthwash products, additional samples were taken from unopened products in the ICU and central storage units. In total we took 20 additional samples from mouthwash solutions. *B. cepacia* was also detected in unopened products. Contamination was detected in all samples (17/17) of a specific batch (G05) of the mouthwash solution. In total, 17/20 of opened and unopened products showed growth. Three unopened solutions without growth had a different batch number (G11). The G05 batch was in use on 24 February 2019 (during the preceding two weeks). The remaining 14 environmental cultures showed no growth other than *Pseudomonas aeruginosa* and *Serratia marcescens from sin*ks. Cultures from hands of the HCW showed no significant growth.

Overall, six patients became colonized, and three of them developed VAP. The median age of colonized patients was 76 (25–75%: 73.5–86). The median time to colonization from admission to ICU was 17.5 days (25–75%: 7.7.5–48.25).

**Fig. 2:**
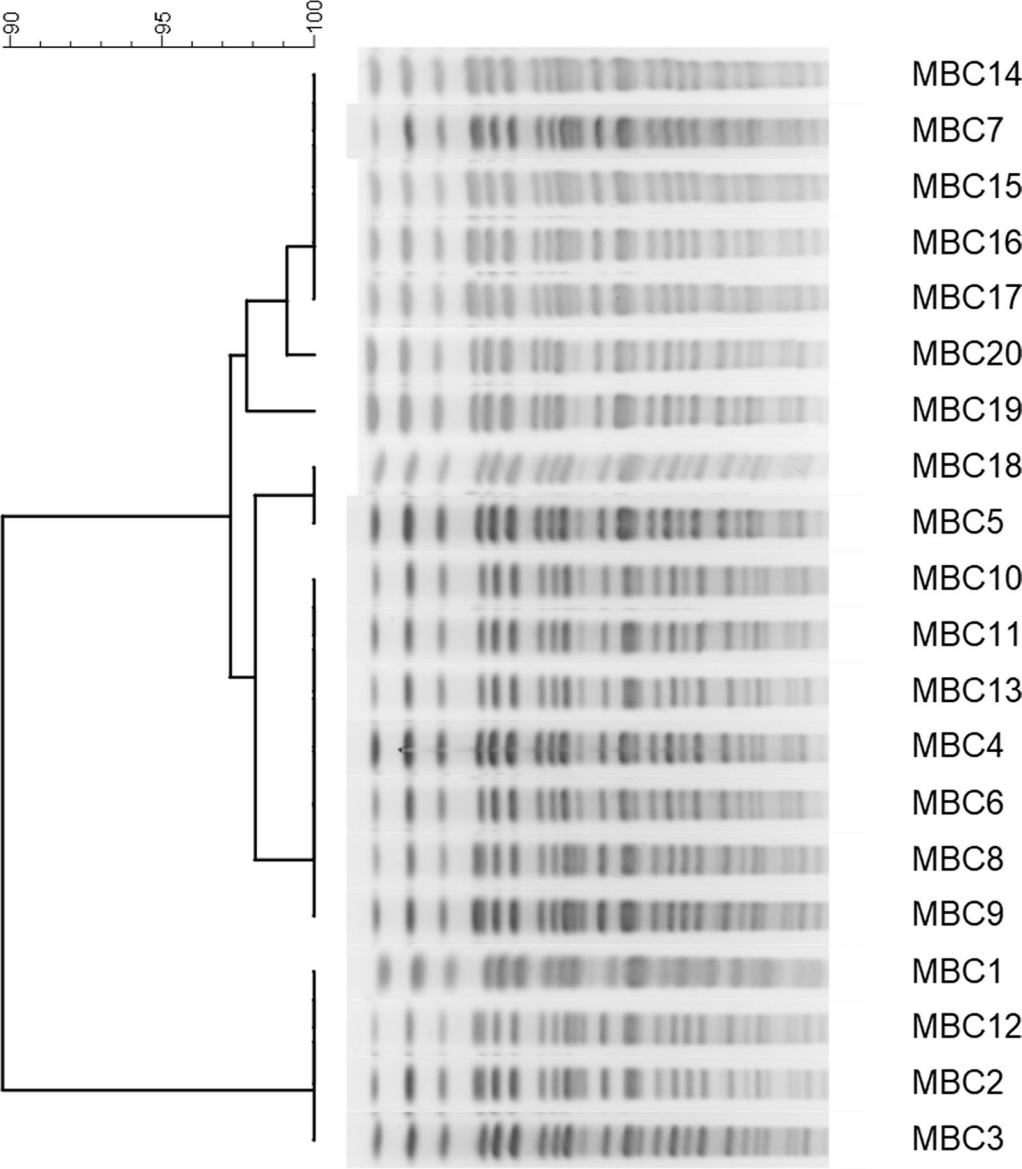
Pulsed-field gel electrophoresis profiles SpeI-digested chromosomal DNA and dendograms from computer-assisted analysis of the profiles. MBC8, 11, 14, 15, 17, and 20 are isolated from the patients’ respiratory samples, and the remaining isolates are from mouthwash solutions

Figure 2 shows the PFGE dendrograms of *B. cepacia* isolates from the patients and the mouthwash solutions. The PFGE detected five pulsotypes out of 20 *B. cepacia* isolates. There was a 90% similarity between the two clusters. The isolates in the second cluster had a 96% similarity.

### Interventions and measures taken

We contacted the neighboring hospitals using the same product. They reported no additional *B. cepacia* cases. We recommended to cohort all colonized patients in the same section of the unit. The use of the product was stopped throughout the institution on the 10th day of the outbreak. IPC monitored the hand hygiene compliance of the unit and gave feedback to HCWs during the outbreak. Infection prevention strategies to prevent HAIs was reminded to the staff. The last case with *B. cepacia* colonization was identified 29 days after the outbreak onset, and no further clusters were identified after discontinuing the contaminated solution.

## Discussion

The investigation revealed a batch of unopened 2% CHG mouthwash solution was the source of this *B. cepacia* outbreak. The PFGE revealed the same strain (90% similarity) that caused the outbreak. The infection control committee took corrective action by the hospital-wide withdrawal of the product. Since the discontinuation of the contaminated solution, we did not detect additional *B. cepacia* infection or colonization. The microbiologic investigation of this outbreak was initiated after the third patient was diagnosed with *B. cepacia* colonization.

Information regarding the role of *B. cepacia* in healthcare-associated infections in Turkey is scarce. Dizbay et al. reported *B. cepacia* infection incidence as 0.26 per 1000 admissions, which accounted for 0.7% of all nosocomial isolates. The most common type of infection was pneumonia. The crude mortality rate of patients with *B. cepacia* complex was 53.8% [8].

*B. cepacia* is clinically relevant in patients with structural lung disease and immunosuppressive patients. If colonized, these patients may develop challenging to treat infections, mostly pulmonary infection. Given its nature of broad antimicrobial and antiseptic resistance, it can survive in medical solutions [9]. It can also cause outbreaks in non-immunocompromised patients due to contaminated medical equipment and solutions [4, 10]. Peterson et al. investigated a clonal outbreak of *B. cepacia* pneumonia in patients without cystic fibrosis. They identified the sink as the source which might have contaminated the respiratory care items [11]. Several studies have reported contamination during manufacturing and after opening the product [2, 12–15]. Shaban et al. reported a nationwide outbreak of *B. cepacia* bacteremia in 2017. They isolated the 11 isolates of *B. cepacia* in 4 hospitals and identified the point source as the contaminated gel packs in sachets used in the sterile ultrasound probe covers [16]. A recent outbreak report showed that contaminated analgesic gel used in urological procedures caused *B. cepacia* bacteremia in nine patients [17].

## Conclusion

Contaminated solutions used in the patient care activities could cause significant outbreaks. This outbreak emphasizes the potential consequences of *B. cepacia* in critical patients, particularly in intensive care units. Prompt and in-depth epidemiological investigation of such clusters is significant for identifying the source of and controlling the outbreak.

## Data Availability

The data is available upon request.

## Abbreviations

CHG: Chlorhexidine
ICU: Intensive care unit
HAI: Hospital-acquired infections
IPC: Infection prevention and control
CDC: Centers for Disease Control
HCW: Healthcare worker
PFGE: Pulsed-field gel electrophoresis
UPGMA: Unweighted Pair Group Method with the mathematical average
VAP: Ventilator associated pneumonia
*B. cepacia*: *Burkholderia cepacia*
